# A Finite Element Study Of the Interaction of Transient Stress Waves With Planar Flaws

**DOI:** 10.6028/jres.092.026

**Published:** 1987-08-01

**Authors:** Mary Sansalone, Nicholas J. Carino, Nelson N. Hsu

**Affiliations:** National Bureau of Standards Gaithersburg, MD 20899

**Keywords:** concrete, finite element analysis, flaw, Green’s function, impact, impact-echo method, nondestructive testing, wave propagation

## Abstract

This paper presents a finite element study of transient wave propagation in plates containing planar flaws. The effects on displacement waveforms caused by waves diffracted from the sharp edges of a flaw are determined. Displacement fields within a plate containing a flat-bottom hole show the interaction of transient stress waves with a planar flaw. Waveforms obtained from the finite element analysis were compared with experimentally obtained waveforms.

## Introduction

A previous paper by the authors [1]^1^ presented a study of transient stress wave propagation in plates. In the previous work, the finite element method was used to study the displacement and stress patterns generated by a transient point load on a plate. Good agreement was found when surface displacement waveforms obtained from finite element analyses were compared with those obtained from the exact Green’s function solutions for impact on an infinite plate. The discussion and results presented in reference [1] form the basis for finite element studies of transient stress wave propagation in solids containing defects—problems for which no exact solutions exist.

This paper presents finite element studies of the interaction of transient stress waves with planar discontinuities in an elastic solid. Emphasis is placed on the effects on displacement waveforms caused by waves diffracted from the edges of a flaw. An aluminum plate with a flat-bottom hole was chosen for the initial finite element studies because its geometry is simple and the physical specimen was easy to fabricate. This allowed the finite element results to be verified by comparison with experimentally obtained waveforms. Displacement waveforms obtained from analyses of planar, disk-shaped flaws in aluminum and concrete plates are presented.

## Background

**Figure 1:**
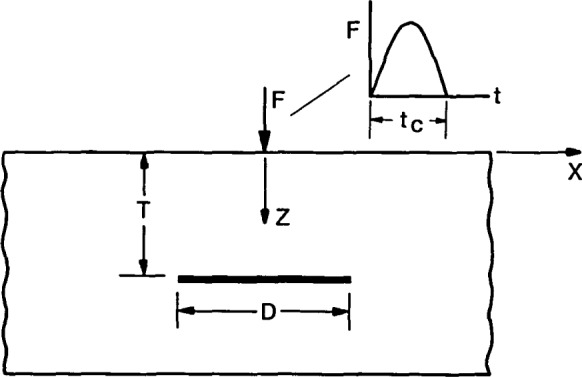
The important variables affecting the response of a planar disk-shaped void in a plate.

Figure 1 shows a schematic representation of point impact on a plate containing a planar disk-shaped flaw. The center of the flaw is located directly under the point of impact and the flaw is parallel to the top surface of the plate. Point impact is generated by dropping a small sphere onto the surface of the plate. The time-history of the contact force generated by the elastic impact of a small sphere on a large plate can be approximated as a half-cycle sine curve (see fig. 1). This is the case that will be discussed in this paper. For this flaw geometry and impact time-history, the important variables affecting the response are the diameter, *D*, and depth, *T*, of the flaw, and the frequency content of the stress waves generated by impact. For a half-cycle sine curve time-history, the frequency content is determined by the contact time, *t*_c_, of the impact [2]. A long contact time produces stress waves made up primarily of large amplitude, low frequency components. Stress waves produced by a short duration impact contain a broader range of frequencies in the waves; however, the amplitude of each component frequency is lower.

Figure 1 also shows the assumed coordinate axis. The positive z-direction will be referred to as the downward direction in this paper; the negative z-direction will be referred to as the upward direction.

**Figure 2:**
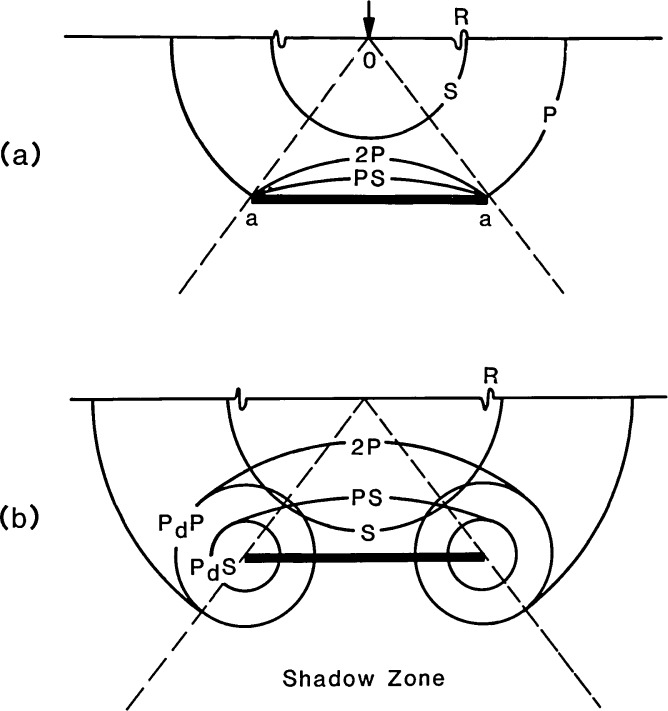
Diffraction at the edges of a crack: a) P-ray incident upon the edges of a crack; and, b) cylindrical wavefronts P_d_P and P_d_S emitted from tips.

When a transient stress wave is incident upon a crack within a solid, the following phenomena occur: specular reflection from the crack face; diffraction at the edges of the crack; and, mode conversion of waves on the crack face and at the edges of the crack. Figure 2(a) shows the specularly reflected P-wavefront (2P) and the mode-converted PS-wavefront produced when a spherical P-wave is incident upon a circular crack. Rays OA, which intersect the edges of the crack, will be diffracted as shown in figure 2(b), producing two diffracted waves, P_d_P and P_d_S. The designation used for diffracted waves includes the incident wave and the wave produced by diffraction. For example, P_d_S is the designation for the diffracted S-wave that is produced by an incident P-wave. The diffracted waves propagate along wavefronts that form a torus of circular cross section; the circumference of the crack is the center of the torus. A similar set of specular (2S), mode-converted (SP), and diffracted waves (S_d_P and S_d_S) are produced when the S-wave is incident upon a crack. The amplitude of particle motion in each of the specularly reflected, mode-converted, and diffracted waves varies with direction and depends upon the size, geometry, and depth of the crack, the frequency content of the waves incident upon the crack, and the orientation of the crack with respect to the propagating waves. As shown in figure 2(b), there is a region beneath the crack called the shadow zone, where direct P- and S-waves cannot penetrate.

## Plate With a Flat-Bottom Hole

In the impact-echo method, a surface displacement waveform recorded near the point of impact is used to obtain information about wave reflection in the interior of the solid. When sharp internal discontinuities exist, the displacements caused by the arrival of diffracted waves are added to the displacements caused by the arrival of reflected waves. Correct interpretation of waveforms requires an understanding of the displacements caused by diffracted waves. In this study, the finite element method is used to gain this understanding. Analyses were performed using DYNA2D [4], a finite element program developed at Lawrence Livermore National Laboratory.

Experimental verification of the results obtained from the finite element analysis was necessary. Thus, a plate containing a flat-bottom hole was used for the initial studies since this test specimen could be easily fabricated.

In the discussion of the elastic response of a plate containing a flat-bottom hole to point impact on the top surface, the following analytical results are presented: 1) the displacement time-history of the surface of the hole directly under the point of impact; 2) displacement fields recorded at various times to show transient stress waves propagating within the plate; and 3) the displacement time-history of a point on the top surface of the plate, near the point of impact. To aid in understanding the effects of diffraction, the displacement time-histories obtained from the plate with a flat-bottom hole are compared to time-histories obtained from a solid plate. Finally, a displacement time-history obtained from a finite element analysis is compared to an experimentally obtained impact-echo displacement waveform.

**Figure 3:**
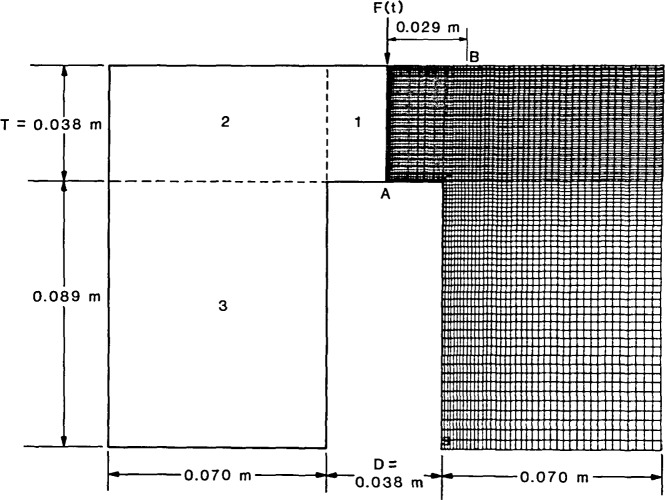
Finite element model of a plate with a flat-bottom hole.

The dimensions of the plate with the flat-bottom hole are shown in figure 3. The diameter, *D*, is 38 mm and the depth to the hole, *T*, is 38 mm; this geometry corresponds to a *D*/*T* value of 1. The axisymmetric, finite element model is shown on the right half of the figure. The plate is made of aluminum and the values of the material properties used in the analysis were: modulus of elasticity equal to 7.1×10^7^ kPa, Poisson’s ratio equal to 0.33, and density equal to 2700 kg/m^3^. These values resulted in P-, S-, and Rayleigh (R) wave speeds of 6242, 3144, and 2930 m/s, respectively.

The axisymmetric finite element mesh was composed of three regions. These regions are shown on the left half of figure 3. The smallest elements are used above and near the edges of the hole. In Region 1, 0.75 mm square elements were used to construct the finite element model. In Regions 2 and 3, the 0.75 mm elements are made gradually larger as the distance from the hole increases.

The time history of the impact loading was a half-cycle sine curve with a 2 μs contact time (*t*_c_=2 μs). An important parameter used to characterize the impact is the ratio of the contact time of the impact, *t*_c_, to the time it takes for the P-wave to propagate down through the plate, be reflected from the surface of the hole, and propagate back to the top surface of the plate, *t*_2P_ [2,3]. If the top of the hole is considered the reflecting interface, *t*_2P_ is equal to 12.2 μs; thus, *t*_c_*/t*_2P_ is equal to 0.16. This small value was chosen so that displacements caused by various waves would be easier to distinguish. The transient load was applied as a uniform pressure over the two elements at the top of the plate adjacent to the centerline of the plate. In the analyses of aluminum plates discussed in this paper, values of displacement and stress were stored in data files every 0.2 μs.

### Displacement Response at the Center of the Hole

**Figure 4:**
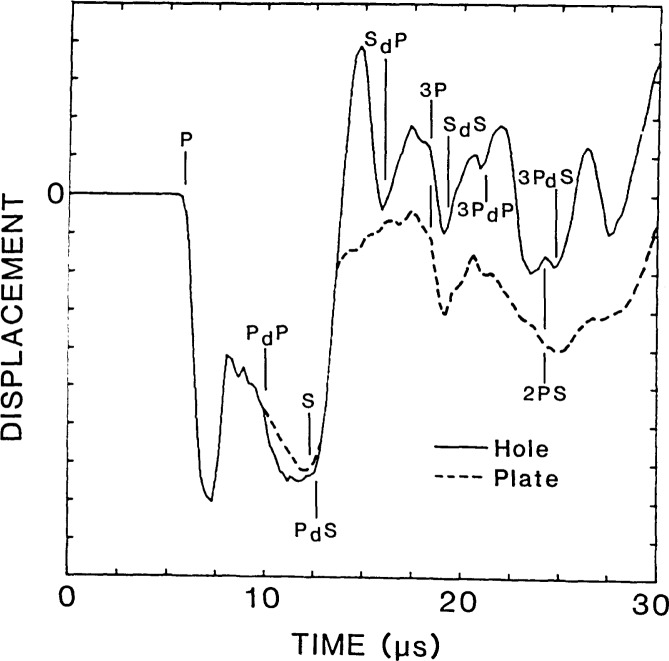
Displacement waveforms at the center of the flat-bottom hole and at the epicenter of a solid plate.

The vertical displacement waveform obtained at the center of the flat-bottom hole (point A in fig. 3) is shown as a solid line in figure 4. This response consists of vertical surface displacements caused by the arrival of the direct waves, multiply reflected waves (3P), mode-converted waves (2PS), and diffracted waves (P_d_P, P_d_S, S_d_P, S_d_S, 3P_d_P, etc.). The computed arrival times of the various waves are indicated on the waveform. The analysis was terminated before waves reflected from the sides and the bottom of the plate arrived at the hole.

For comparison, the dashed line in figure 4 shows the response of a point at the center of the bottom surface of a solid aluminum plate. The plate was 38 mm thick and was subjected to the same 2 μs duration impact used in the analysis of the plate containing the flat-bottom hole. The finite element mesh for the plate was comprised of Regions 1 and 2 of the mesh shown in figure 3; that is, the elements below the top surface of the hole were eliminated. The vertical surface displacement for the plate consists of displacements caused by direct, multiply reflected, and mode-converted waves. (In ref. [1] the plate response was discussed in detail.) The waveform obtained from the flat-bottom hole (solid line in fig. 4) is the superposition of this plate response and the displacements caused by diffracted waves. Thus, the differences between the waveforms shown in figure 4 are due to the displacements caused by diffracted waves from the edges of the flat-bottom hole.

Using the waveform obtained from the plate (dashed line) as a baseline response, that is, the component of the response common to both waveforms, the effect of the diffracted waves on the displacement pattern can be determined. The arrival of diffracted wave P_d_P produces a noticeable change in the displacement pattern, but causes only a slight increase in the magnitude of the displacement compared with the baseline response. The P_d_S-wave causes an upward displacement of the surface which pushes the surface of the hole well above its original undisturbed position. The S_d_P-wave also produces an upward displacement of the hole; however, the magnitude of this displacement is less than that produced by P_d_S. The S_d_S-wave causes an increase in the magnitude of the upward displacement that occurs after the 3P-wave. Arrivals of subsequent diffracted waves (3P_d_P and 3P_d_S) produce noticeable displacements in the waveform obtained from the hole (solid line) that are absent in the waveform obtained from the plate (dashed line). Thus, for this particular flat-bottom hole geometry and for the 2 μs duration impact used in the analysis, relatively large amplitude diffracted waves are produced which significantly alter the displacement pattern obtained at the center of the hole compared with that obtained at the bottom of a solid plate.

### Displacement Fields

**Figure 5:**
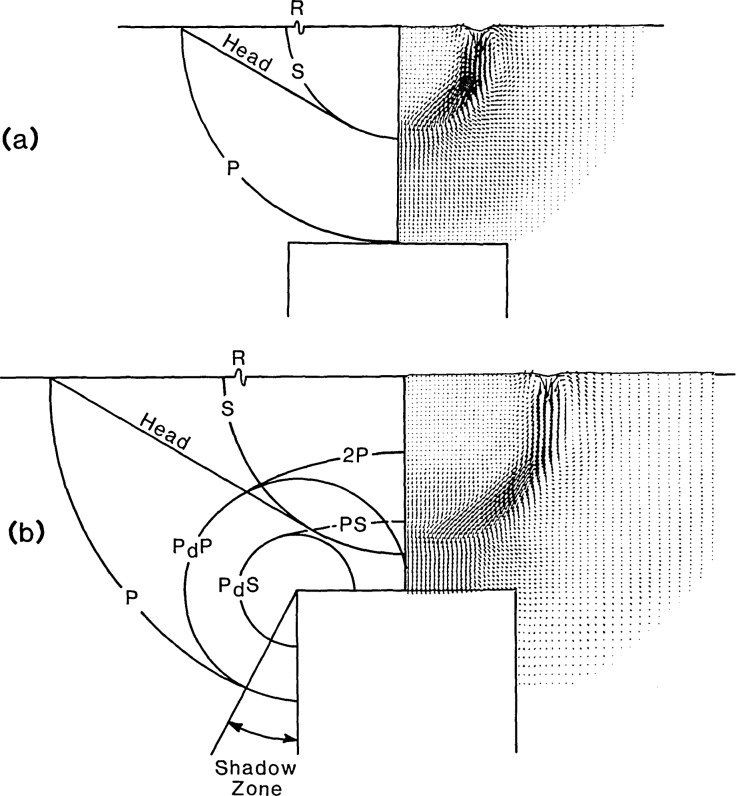
Vector plots of the displacement fields in a plate with a flat-bottom hole: a) 6.1 μs after the start of impact; and b) 10 μs after the start of impact.

The right sides of (a) and (b) of figure 5 show vector plots of the displacement fields in the plate containing the flat-bottom hole at 6.1 and 10 μs, respectively, after the start of the impact. (Since the displacement field is axisymmetric, the field for only half of the specimen is shown.) The corresponding positions of the P-, S-, mode-converted, and diffracted wavefronts are indicated on the left hand side of each figure.

The displacement field at 6.1 μs [fig. 5(a)] shows the P-wavefront arriving at the hole. The S-wavefront has traveled approximately half the distance to the hole. The pattern of displacements between the P- and the S-waves, and the P-wake, which was discussed in ref. [1], is evident.

Figure 5(b) shows the displacement field at 10 μs. The reflected P-wave (2P) is evident. The mode-converted PS-wave, produced by the incident P-wave, is difficult to distinguish as it overlaps the S-wave. The surface of the hole is displaced downward due to the displacements caused by the preceeding P-wave and the P-wake, which at 10 μs is incident upon the hole (see fig. 4). The pattern of displacements trailing the S-wave, the S-wake is evident (see ref. [1]). The low amplitude head wave can also be distinguished. Diffracted waves P_d_P and P_d_S have been produced by the P-wave incident at the edge of the hole. The P_d_P-wave is clearly evident in the shadow zone behind the hole (fig. 5(b)) where the direct P-wave cannot penetrate. The pattern of displacements caused by the P_d_S-wave is easy to distinguish.

**Figure 6:**
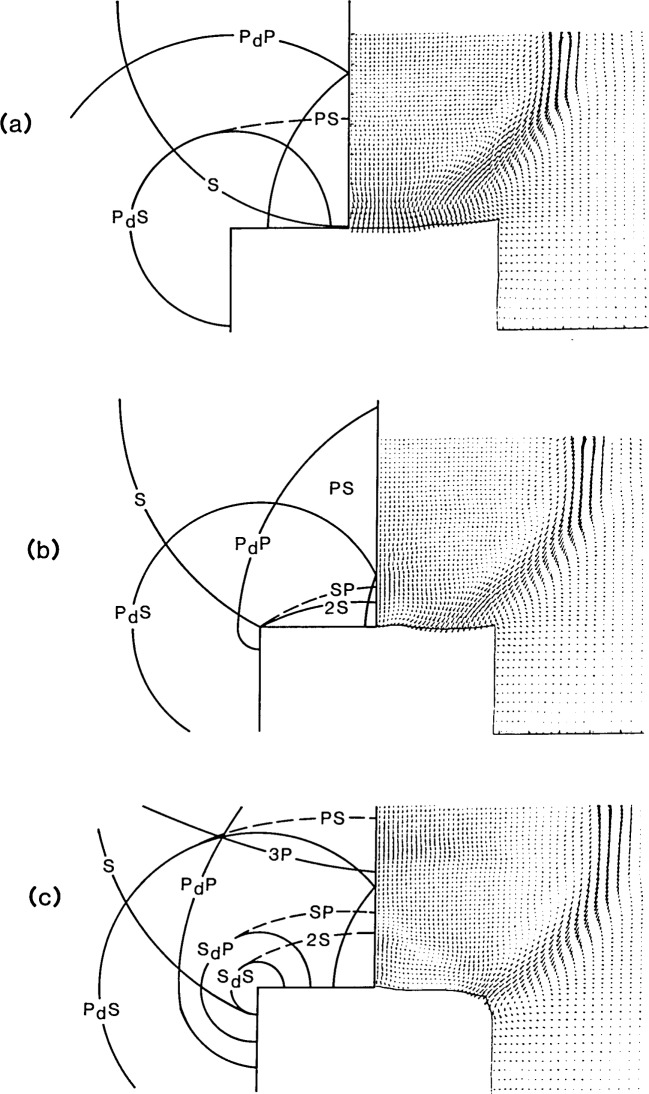
Close-ups of vector plots of displacement fields around the flat-bottom hole at various times after the start of impact: a) 12 μs; b) 13.5 μs; and c) 15 μs.

To show the effects of the diffracted waves on the displacement response at the hole, close-ups of the region near the hole are shown on the right side of figures 6(a) through 6(c). These vector plots represent the displacement fields at 12, 13.5, and 15 μs after the start of the impact. The positions of the wavefronts in this region are shown on the left side of each figure.

In the displacement field at 12 μs (fig. 6(a)), the S-wave is incident on the center of the hole. The P_d_P-waves are overlapping in the region above the hole.

Figure 6(b), the displacement field at 13.5 μs, shows the S-wave incident upon the edge of the hole. In the radiation pattern of the S-wave [1], vertical displacements in the center of the plate are very small; therefore, the S-wave does not cause significant downward movement of the center region of the hole. Thus, at 13.5 μs the center region of the hole is recovering and moving upward. At larger angles in the radiation pattern, the vertical component of the displacement in the S-wave increases. Thus, the outer region of the hole is depressed by the incident S-wave. The fronts of diffracted waves P_d_S (one from each edge) have just arrived at the center of the hole. Recall that in the displacement waveform shown by the solid line in figure 4, it was P_d_S that produced the large upward displacement that pushed the center of the hole well above its original undisturbed position. In figure 4, at 13.5 μs the center of the hole is moving rapidly upward, but it is still displaced below its undisturbed position.

At approximately 15 μs the upward displacement of the center of the hole has reached its maximum (see fig. 4). The center of the hole is displaced upward, while the edge of the hole is pushed down by the incident S-wave. The diffraction pattern has become complicated; at 15 μs four diffracted wavefronts have been produced by the incident P- and S-waves. Each of these diffracted waves will give rise to new diffracted waves when they reach the opposite edge of the hole. These doubly diffracted wavefronts appear to be of secondary importance. The arrival of multiple reflected P- and S-waves from the top surface of the solid, such as the 3P-wave [seen at the top of fig. 6(c)], will also produce diffracted waves. In addition, diffracted waves will be reflected from the top surface of the plate giving rise to new diffracted waves when they strike the edge of the hole.

### Impact-Echo Response

Displacement responses recorded on the top surface of a plate are referred to as impact-echo waveforms. Impact-echo waveforms obtained from both the plate with the flat-bottom hole and the solid plate can be used to determine the effects caused by diffracted waves on the displacement response at the top surface. The effects caused by diffracted waves can be more difficult to determine at the top surface of the plate than at the center of the hole, because surface waves often mask the effects caused by the specularly reflected and diffracted waves. However, since the relative wave arrival times of surface waves, reflected waves, and diffracted waves differ at increasing distances from the impact point, the problem can be overcome by studying waveforms obtained at different points along the surface.

**Figure 7:**
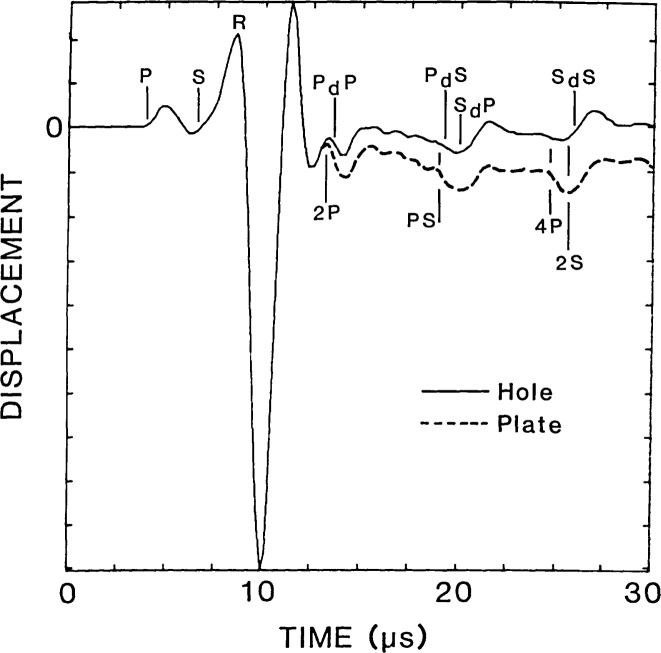
Impact-echo displacement waveforms from a plate with a flat-bottom hole and from a solid plate.

A finite element study of impact-echo waveforms obtained from the plate with the flat-bottom hole showed that P_d_P, P_d_S, S_d_P, and S_d_S all cause upward movement of the top surface of the specimen. This is in contrast to the downward displacements caused by specularly reflected P-waves. Displacement waveforms represent the superposition of displacements caused by specularly reflected and diffracted waves. Thus, depending upon the arrival times of the diffracted waves relative to the specularly reflected waves, the effect of a diffracted wave can be to lessen or increase the displacements caused by specularly reflected waves. As an example, the solid line in figure 7 shows a displacement waveform obtained on the top surface of the plate containing the flat-bottom hole. The separation, H, between the impact point and the point where the response was recorded was 25 mm (point B in fig. 3). The response consists of displacements caused by the arrivals of direct P-, S-, and R-waves propagating along the surface, reflected P- and S-waves (2P, 2S, 4P), mode-converted waves (PS), and diffracted waves (P_d_P, P_d_S, S_d_P, S_d_S, etc.). The arrival times of these various waves are indicated on the waveform. In the figure, arrival times of diffracted waves correspond to the arrival of waves diffracted from the point on the edge of the hole nearest the receiver; these diffracted waves produce the most significant effects in the displacement response.

For comparison, the dashed line in figure 7 shows an impact-echo response that was obtained from the analysis of the 38 mm thick plate subjected to a 2 μs duration impact. As for the plate containing the flat-bottom hole, the separation between the impact point and point where the waveform was recorded was 25 mm. In the portion of the waveform after the R-wave signal, the response is dominated by the arrival of P-waves, which cause downward displacements; the top surface is never displaced above its undisturbed position.

From a comparison of the waveforms in figure 7, it is concluded that the diffracted waves cause the following effects: P_d_P noticeably reduces the downward displacement caused by the 2P-wave; P_d_S appears to have little effect, although it slightly reduces the downward displacement caused by the PS-wave; S_d_P and S_d_S both cause upward displacements.

If another point on the top surface of the plates had been chosen for this comparison, the displacement response caused by superposition of the specular and diffracted waves would be different, since both the arrival times and the amplitudes of the waves change relative to one another.

### Comparison to Experimentally Measured Waveform

**Figure 8:**
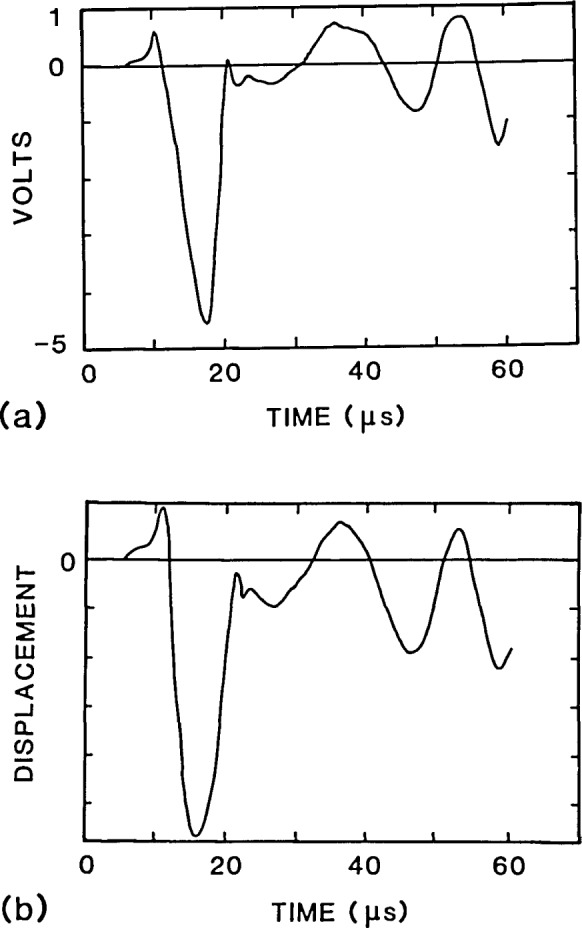
Comparison of analytical response with experimentally obtained response for a plate with a flat-bottom hole: a) experimental waveform; and, b) finite element waveform.

No exact solutions are available to compare with the finite element displacement waveforms; therefore, the finite element solution was compared to an experimentally obtained waveform. An impactecho test was carried out on an aluminum plate containing a flat-bottom hole with dimensions as shown in figure 3. The impact point was located at the center of the specimen, and the spacing between the impact and receiving transducer was 29 mm. The impact source was a 1.6 mm diameter steel ball that was dropped from a height of 30 mm. The contact time of the impact was 10 μs. A broadband displacement transducer, developed at NBS for acoustic emission testing [5], was used as the receiver. This transducer is composed of a small conical PZT sensing element attached to a large brass cylinder, and has a uniform frequency response up to 1 MHz. The output of this transducer is proportional to normal surface displacement. The small 1.5 mm diameter of the conical tip of the transducer approximates a point receiver. Figure 8(a) shows the experimental waveform up to a time of 60 μs. The waveform includes reflections from the sides and bottom of the plate. At 40 μs the P-wave reflection from the bottom of the plate arrives at the receiver causing a large downward displacement; at 52 μs the R-wave reflected from the side of the plate arrives at the receiver also causing a large downward displacement.

Figure 8(b) shows a displacement waveform obtained from a finite element analysis of the plate containing the flat-bottom hole subjected to an impact having a force-time history in the shape of a half-cycle sine curve with a contact time of 10 μs. The spacing between impact point and the point where the displacement was recorded was also 29 mm.

There is good agreement between the experimental response and the response predicted by the finite element analysis. The 10 μs duration impact results in a *t*_c_/*t*_2P_ value of approximately 0.8. For this contact time and for the spacing between the impact point and the receiver, effects due to individual wave arrivals are difficult to discern [3]. The superposition of effects causes the arrival of the initial internal reflections to be hidden in the response of the surface to the large amplitude R-wave. However, the important feature in both waveforms is that effects caused by diffracted waves move the surface above its undisturbed position.

This comparison established the validity of using the finite element method for modeling transient wave propagation in elastic solids containing flaws. Thus, the finite element method can be used to study the interaction of transient waves with flaw geometries more likely to be encountered in actual materials. In the following section, the discussion of a plate containing a flat-bottom hole is extended to study a similar, but more realistic, geometry—a plate containing a planar, disk-shaped flaw.

## Planar Disk-Shaped Flaws in Plates

### Flaw in Aluminum

The dimensions of an aluminum plate containing a disk-shaped flaw are identical to those shown in figure 3 for the plate containing the flat-bottom hole. The depth to the top surface of the flaw and the diameter of the circular flaw are identical to the depth to the top surface of the hole and the diameter of the flat-bottom hole. The finite element model was created by using the mesh shown in figure 3 with the addition of the elements required to form an interior disk-shaped void from the hole. The void was 1 mm thick. The analysis was carried out using the same material properties and loading conditions as in the analysis of the plate with the flat-bottom hole.

**Figure 9:**
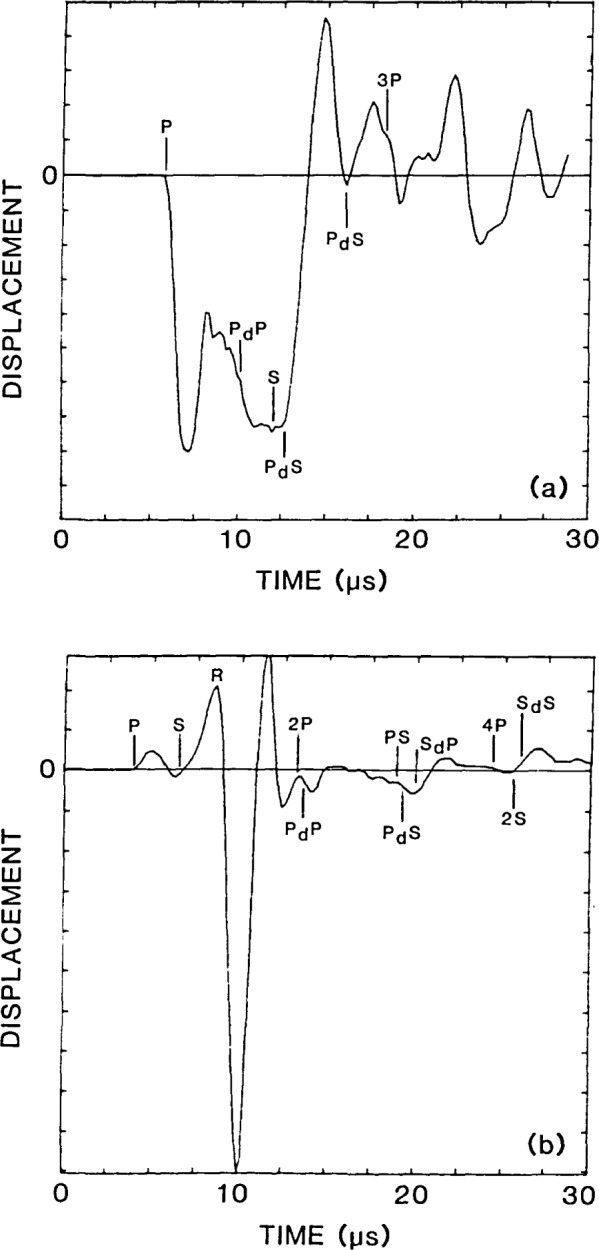
Planar disk-shaped flaw in an aluminum plate: a) displacement at the center of the top surface of the flaw; and, b) displacement at a point on the top surface of the plate.

Displacement waveforms obtained at the center of the top surface of the flaw (point A in fig. 3) and at a point on the top surface of the plate, 25 mm from the point of impact (point B in fig. 3), are shown in figures 9(a) and (b), respectively. Wave arrival times are indicated on both waveforms. These waveforms do not need detailed explanation as they are very similar to those obtained from the plate containing the fiat-bottom hole [figs. 4(a) and 7(a)]. A comparison of the waveforms obtained at the surfaces of the hole and the disk-shaped flaw shows that there are minor differences in the responses which occur after the arrival of the S_d_S-and 2PS-waves. At the top surface the waveforms are almost identical, except that the displacements obtained from the plate containing the disk-shaped flaw are shifted slightly above those obtained from the plate with the flat-bottom hole. Thus, it appears that the presence of the material below the flaw does not significantly change the overall response for the period of time prior to wave reflections from the bottom of the plate.

The displacement patterns that have been discussed were obtained for planar flaws in aluminum. Aluminum has a Poisson’s ratio of 0.33 which results in a ratio of S- to P-wave speeds of 0.50. As mentioned, aluminum was used so that waveforms obtained from the finite element analysis could be compared to waveforms obtained from an easily fabricated test specimen. However, the primary focus of the research program is in applying the impact-echo method to concrete, a material with a Poisson’s ratio of approximately 0.2 in the elastic range. For a Poisson’s ratio of 0.2, the S-wave speed is 61% of the P-wave speed; this ratio is significantly different from that in aluminum. This difference affects the relative arrival times of the various wavefronts. For comparison, the displacement patterns obtained from a finite element analysis of a planar disk-shaped flaw in a plate having elastic properties representative of concrete are presented.

### Flaw in Concrete

An analysis was performed for a planar, disk-shaped void in a 0.5 m thick concrete plate. The diameter and the depth of the flaw are both 0.2 m resulting in a *D/T* value equal to 1, the same *D/T* value as in the analyses of the flaws in aluminum. The concrete was modeled as a linearly-elastic, homogeneous solid, with the following material properties: modulus of elasticity of 3.31×10^7^ kPa, Poisson’s ratio of 0.2, and a density of 2300 kg/m^3^. These values result in P-, S-, and R-wave speeds of 4000, 2440, and 2240 m/s, respectively. In the Region 1, 5 mm square elements were used in the finite element model. The time history of the impact load was a half-cycle sine curve with a 16 μs contact time, which gave the desired ratio of *t*_c_*/t*_2P_ equal to 0.16.

**Figure 10:**
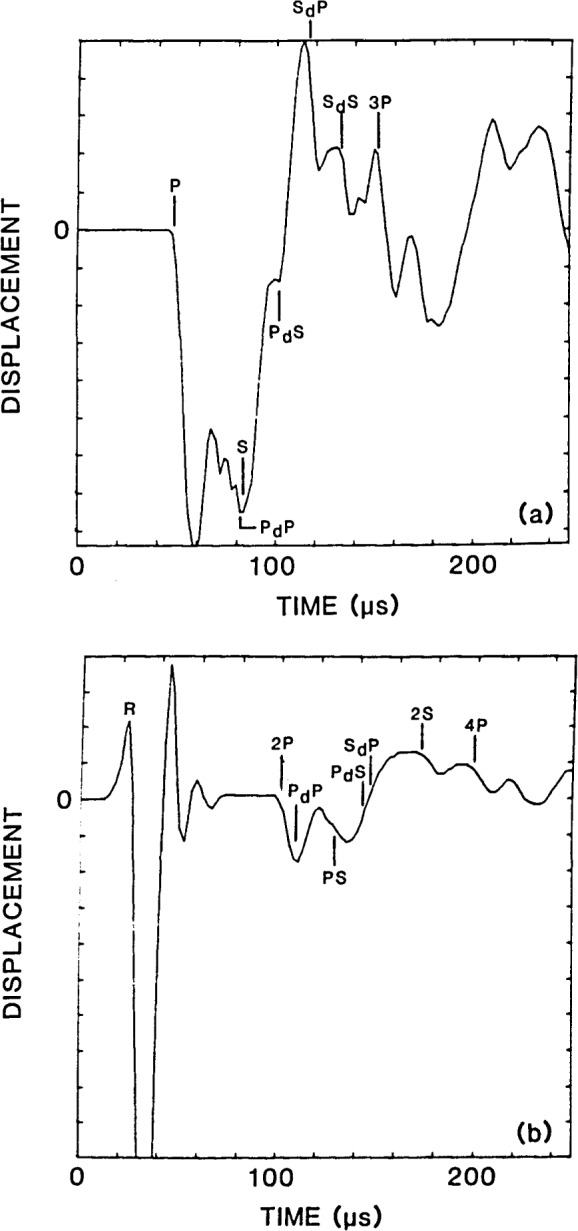
Planar disk-shaped flaw in a concrete plate: a) displacement at center of the top surface of the flaw; and, b) displacement at a point on the top surface of the plate.

Displacement waveforms obtained at the center of the top surface of the flaw and at a point on the top surface of the plate, 50 mm from the impact point, are shown in figures 10(a) and (b), respectively. Wave arrival times are indicated on the waveforms. The displacement caused by each wave is the same as has been discussed; however, since there is less of a difference between the P-and S-wave speeds, individual wave arrivals in concrete are more closely spaced than in aluminum. Thus, the displacement waveforms obtained from the concrete specimen are different from those previously shown for the same flaw in aluminum. For example, compare the displacement pattern in figure 9(a) with that in figure 10(a). In the concrete specimen, the P_d_S-wave arrives somewhat later than the PS-wave, rather than at approximately the same time as in the aluminum plate; thus, the large upward displacement caused by the P_d_S-wave is easily identified in figure 10(a). As in aluminum, if the response obtained from the concrete plate containing the disk-shaped flaw is compared to the response obtained from a solid concrete plate [such as in fig. 5 of ref. [1]], the effects caused by diffracted waves are clearly evident. Diffracted waves produce more frequent fluctuations in the displacement and they move the top surface of the flaw above its undisturbed position. For the top surface displacement shown in figure 10(b), the most noticeable effect due to diffracted waves is the large upward surface displacement caused by the arrivals of the P_d_S- and S_d_P-waves.

In figure 10(b), there is a period after the arrival of the R-wave when the displacement appears to oscillate about its undisturbed position. These oscillations are due to numerical ringing of the finite elements which occurs due to rapid changes in displacement [1].

## Summary

A previous finite element study of the displacement and stress fields created by a transient point load on an elastic solid [1] served as the basis for the present study of the displacement fields created by the interaction of transient stress waves and planar flaws in solids. The elastic responses produced by surface impact on aluminum and concrete plates containing flat-bottom holes and planar disk-shaped flaws were studied. Displacement waveforms obtained from the plates were compared to waveforms obtained from solid plates to determine how displacement patterns are affected by the waves created by diffraction at the sharp edges of a discontinuity. It was shown that the displacement waveforms obtained from a plate with a flat-bottom hole are very similar to those obtained from a plate containing a planar disk-shaped flaw.

In this and the previous paper, comparisons of finite element waveforms to exact and experimentally obtained displacement waveforms established the validity of using finite element analysis for the study of transient wave propagation in elastic solids. These papers have laid the groundwork for using the finite element method to study elastic solids of arbitrary geometry and to study solids containing a variety of types of flaws—problems for which no exact solutions exist.

## Footnotes

1 Figures in brackets indicate literature references.
